# Multi-Locus Microsatellite Typing of Colonising and Invasive *Aspergillus fumigatus* Isolates from Patients Post Lung Transplantation and with Chronic Lung Disease

**DOI:** 10.3390/jof10020095

**Published:** 2024-01-24

**Authors:** Joshua D. Birnie, Tanveer Ahmed, Sarah E. Kidd, Glen P. Westall, Gregory I. Snell, Anton Y. Peleg, Catherine Orla Morrissey

**Affiliations:** 1 University Hospital Geelong, Barwon Health, Geelong, VIC 3220, Australia; joshuabirnie@gmail.com; 2 Department of Infectious Diseases, Alfred Health and Monash University, Melbourne, VIC 3004, Australia; t.ahmed@alfred.org.au (T.A.); anton.peleg@monash.edu (A.Y.P.); 3 National Mycology Reference Centre, SA Pathology, Adelaide, SA 5000, Australia; sarah.kidd@sa.gov.au; 4 Lung Transplant Service, Department of Respiratory Medicine, Alfred Health and Monash University, Melbourne, VIC 3004, Australia; g.westall@alfred.org.au (G.P.W.); g.snell@alfred.org.au (G.I.S.); 5 Infection and Immunity Program, Monash Biomedicine Discovery Institute, Department of Microbiology, Monash University, Clayton, VIC 3168, Australia

**Keywords:** *Aspergillus fumigatus*, invasive aspergillosis, colonisation, genotyping, lung transplant, chronic obstructive pulmonary disease, cystic fibrosis, chronic lung allograft dysfunction

## Abstract

*Aspergillus fumigatus* can cause different clinical manifestations/phenotypes in lung transplant (LTx) recipients and patients with chronic respiratory diseases. It can also precipitate chronic lung allograft dysfunction (CLAD) in LTx recipients. Many host factors have been linked with the severity of *A. fumigatus* infection, but little is known about the contribution of different *A. fumigatus* strains to the development of different phenotypes and CLAD. We used multi-locus microsatellite typing (MLMT) to determine if there is a relationship between strain (i.e., genotype) and phenotype in 60 patients post LTx or with chronic respiratory disease across two time periods (1 November 2006–31 March 2009 and 1 November 2015–30 June 2017). The MLMT (STR*Af*) assay was highly discriminatory (Simpson’s diversity index of 0.9819–0.9942) with no dominant strain detected. No specific genotype–phenotype link was detected, but several clusters and related strains were associated with invasive aspergillosis (IA) and colonisation in the absence of CLAD. Host factors were linked to clinical phenotypes, with prior lymphopenia significantly more common in IA cases as compared with *A. fumigatus*-colonised patients (12/16 [75%] vs. 13/36 [36.1%]; *p* = 0.01), and prior *Staphylococcus aureus* infection was a significant risk factor for the development of IA (odds ratio 13.8; 95% confidence interval [2.01–279.23]). A trend toward a greater incidence of CMV reactivation post-*A. fumigatus* isolation was observed (0 vs. 5; *p* = 0.06) in LTx recipients. Further research is required to determine the pathogenicity and immunogenicity of specific *A. fumigatus* strains.

## 1. Introduction

*Aspergillus fumigatus* is a ubiquitous saprophytic filamentous fungus which is opportunistic, causing infection in the immunocompromised and those with chronic lung disease [1,2]. It causes aspergillosis, an overarching term for the spectrum of infection, which ranges from allergic through colonisation to invasive, even in those with the same underlying disease [1]. Lung transplant (LTx) recipients commonly develop colonisation (20–50%), which can be transient, persistent, or progress to invasive disease; however, less frequently (3–15%), *de novo* invasive disease can also develop, despite near uniform immunosuppression [3,4]. Similarly, patients with chronic obstructive pulmonary disease (COPD) can develop colonisation (13–29%), aspergilloma (saprophytic colonisation), semi-invasive chronic cavitary pulmonary aspergillosis, or invasive aspergillosis (IA) (1.3–3.9%) [5,6,7]. Whilst cystic fibrosis (CF) patients commonly develop colonisation (30–80%) they can also develop allergic bronchopulmonary aspergillosis (ABPA) and bronchitis (9–10%) [8,9,10,11,12].

Whilst host immune function is critical to the development of allergic, colonisation, or invasive disease, there are data to indicate that genetic variations between different *A. fumigatus* strains also play a role. Mondon et al. examined the variation in the virulence of different strains of *A. fumigatus* in a mouse model of IA [13]. Using random amplified polymorphic DNA typing, clinical strains (n = 7) and environmental strains (n = 4) were examined to detect the 0.95 kb virulence fragment (EMBL/GenBank accession number: L35210) [13]. These isolates were subsequently used to infect mice, and the clinical isolates with the 0.95 kb virulence fragments resulted in significantly higher mortality rates in the mice than those without them (*p* < 0.02) [13].

Several genotyping methods exist, but multi-locus microsatellite typing (MLMT) is amongst the most discriminatory and reproducible for *A. fumigatus.* In addition, the generated data is easily archivable and portable to allow for longitudinal and inter-laboratory comparisons [14,15,16]. Data from MLMT studies indicate that it can identify certain genotypes associated with certain clinical manifestations (or phenotypes). de Valk et al. identified several different patterns of *A. fumigatus* colonisation after examining 204 isolates sequentially collected from 36 CF patients. These patterns ranged from persistent colonisation by a single identical or closely related strain over a prolonged period (17%) to transient or occasional colonisation with several different strains (36%) [17]. Vanhee et al. examined 256 *A. fumigatus* isolates from eight CF patients and found that all eight patients were colonised with multiple strains but that in 7/8 (87.5%), only certain strains were persistently isolated [18]. Persistent colonisation is a known risk factor for invasive disease [4]. Only a single genotype was found at distant sites, such as the heart, kidney and spleen; however, multiple genotypes were found in the respiratory tracts of 15 haematology patients with proven disseminated IA [19]. This indicates that one genotype becomes dominant and invades the surrounding lung tissue and from there disseminates to other organs.

*Aspergillus* has been implicated in the development of chronic lung allograft dysfunction (CLAD). It is postulated that *Aspergillus*-associated CLAD occurs due to the fungus interacting with the allograft cells, stimulating host–chemokine production, leading to leukocyte recruitment and mononuclear infiltration of the graft tissue. In addition, fibroproliferation and disruption of tissue repair occurs, resulting in progressive occlusion of the airways, leading to a persistent and progressive decline in lung function [20,21,22]. CLAD affects up to 50% of LTx recipients within five years of transplantation and is the single biggest obstacle to long-term survival. A multicentre study of 780 LTx recipients found that the development of CLAD was significantly greater in those patients who had previously isolated small conidia-producing *Aspergillus* spp. (≤3.5 µm) as compared with large conidia-producing *Aspergillus* spp. (Hazard ratio [HR] 1.44; 95% confidence interval [CI] 1.14–1.82; *p* = 0.02) [23]. This lends weight to the theory that certain *Aspergillus* strains are associated with certain clinical phenotypes.

We hypothesised that some strains/genotypes of *A. fumigatus* isolates are more likely to be associated with certain clinical phenotypes. The aim of this study was to genotype *A. fumigatus* complex isolates from patients post LTx or with chronic respiratory disease and correlate with detailed clinical data to determine any relationship between genotypes, IA, colonisation, and/or CLAD.

## 2. Materials and Methods

### 2.1. Study Isolates and Patient Population

All *A. fumigatus* isolates used in this study were collected from patients post LTx or with chronic respiratory disease. Written informed consent was obtained from each patient to genotype the isolates and collect their associated clinical data. The study was approved by Alfred Health Human Research Ethics Committee (184/07, 041/13; amended 15 February 2016). Only the first *A. fumigatus* isolate for each patient was tested in this study.

The 60 isolates tested as part of this study included 30 collected from bronchoalveolar lavage fluid (BAL) or sputum of patients post LTx or with chronic respiratory disease and 30 collected from the BAL of LTx recipients who attended Alfred Health, Melbourne, Australia, between 1 November 2006 and 31 March 2009 and between 1 November 2015 and 30 June 2017, respectively. The two time periods were chosen to determine the influence of any changes in transplant practice on clinical outcomes and to detect any confounding related to shifts over time in the *Aspergillus fumigatus* population (overall).

### 2.2. Isolation of Genomic DNA from A. fumigatus Cultures

We first confirmed that the selected isolates were *A. fumigatus sensu stricto*. For this, conidia from each of the 60 selected isolates were grown on SCG (Sabouraud agar with chloramphenicol and gentamicin) plates for 3–10 days at 30 °C. A 0.1% TWEEN 20 solution (Sigma-Aldrich, St. Louis, MI, USA) (1 mL) was decanted onto the culture plates. Several colonies were gathered into this solution and then transferred to a 2 mL Eppendorf tube (Sigma-Aldrich, North Ryde, NSW, Australia). Genomic DNA was isolated using the MasterPure Yeast DNA Purification Kit (Epicentre, Madison, WI, USA) with modifications to the extraction protocols of Jin et al. [24]. Briefly, the tubes were centrifuged at 13,000 rpm for 10 min (min) and the supernatant discarded. The pellets were then washed with 500 µL of sterile distilled water, centrifuged, and the supernatant discarded. This was repeated using 500 µL of magnesium chloride (MgCl_2_) to wash the pellets.

Next, 450 μL of yeast cell lysis solution was added to each sample, then vortexed for 10 s (s). The tubes were then incubated for 1 h (h) at 65 °C. Following this, the tubes were chilled on ice for 5 min. Next, 225 μL of protein precipitation reagent was added to each sample and the tubes were vortexed for 5 s and centrifuged for 15 min at 16,000× *g* to pellet the cellular debris. The supernatant was transferred to a clean tube and the centrifugation step was repeated. The resulting supernatant was transferred to a clean tube (approximately 500 μL). Then, 500 μL (or an equal volume) of isopropanol was added, mixed by inversion to precipitate the DNA, and pelleted by centrifuging for 15 min at 16,000× g. The supernatant was discarded by pipetting. The DNA pellet was washed in 500 μL of 70% ethanol and centrifuged for 15 min at 16,000× *g*. Any remaining ethanol was removed by pipetting and brief centrifuging. The pellet was allowed to dry before being suspended in 35 μL of TE buffer and treated with 2 μL of RNAase A. The DNA concentration and quality (A_260_/A_280_) were checked using a Nanodrop spectrophotometer. Working concentrations of all DNA samples (10 ng/μL) were made up for use in the PCR step (See Section 2.3).

### 2.3. Molecular Identification of Isolates

Partial calmodulin (CaM) gene region sequencing was performed on the extracted genomic DNA for each isolate, as previously described for fungal identification [25]. Briefly, partial calmodulin gene amplification was performed using CMD5 (5′-CCG AGT ACA AGG ARG CCT TC-3′) and CMD6 (5′-CCG ATR GAG GTC ATR ACG TGG-3′) primers, as previously described [26]. The PCR reaction mix consisted of 1× GeneAmp PCR Buffer II (Applied Biosystems Inc., Foster City, CA, USA), 1.5 mM MgCl_2_, 0.2 mM deoxynucleoside triphosphates (dNTPs) (Bioline Pty. Ltd., Sydney, NSW, Australia), 2.5 U FastStart *Taq* DNA polymerase (Roche Diagnostics Corp., Indianapolis, IN, USA), 0.6 µM of each primer and 20 ng of genomic DNA to a total volume of 25 µL. The following thermocycling conditions were used: initial denaturation at 95 °C for 10 min, followed by 38 cycles of 95 °C for 30 s, 55 °C for 30 s and 72 °C for 1 min, with a final extension step of 7 min.

PCR products were visualised on a 1.5% agarose gel using either a 1 kb or 2 kb ladder (Roche Diagnostics Corp.) to estimate the sizes of the bands. The products were purified using the High Pure PCR Product Purification Kit (Roche Diagnostics Corp.) and sent for Sanger sequencing at the Micromon Sequencing Facility (Monash University, Clayton, VIC, Australia), where the BigDye Terminator v3.1 Cycle Sequencing Kit (Applied Biosystems) was used. Sequence quality was then checked and edited using the Sequence Scanner software v2.0 (Applied Biosystems), and the sequence was then aligned against sequences for type material in the National Center for Biotechnology (NCBI) Basic Local Alignment Search Tool (BLAST) to assess whether they matched to *A. fumigatus* reference strains or to cryptic species within *A. fumigatus* complex.

### 2.4. Microsatellite Genotyping

Genotyping was performed on all isolates by amplifying nine short tandem repeat (STR) regions by three multiplex PCRs, M2, M3 and M4, as previously described (see Table S1 for primers) [16], which amplify di-, tri- and tetra-nucleotide repeat markers, respectively. 6FAM, VIC and NED fluorescent labels were included on forward primers to distinguish intra-PCR amplicons. Amplifications for the M2 multiplex PCR were performed in 1× GeneAmp PCR Buffer II (Applied Biosystems), 1.5 mM MgCl_2_, 0.2 mM dNTPs (Bioline, Memphis, TN, USA), 2.5 U *Taq* polymerase (Roche), 1 µM of each primer and 1 ng of genomic DNA to a total volume of 25 µL. M3 and M4 multiplex PCRs were performed in identical amplification mixes as M2, except the total MgCl_2_ concentration was 3 mM. PCR cycling conditions for each multiplex PCR included an initial denaturation step at 95 °C for 10 min, followed by 30 cycles of 95 °C for 30 s, 60 °C for 30 s and 68 °C for 1 min, with a final extension step of 68 °C for 10 min.

Following PCR, fragment length analysis was performed by the Australian Genome Research Facility (Melbourne, VIC, Australia) using capillary electrophoresis and the AB GeneMapper software Version 5 (Applied Biosystems). STR numbers are based on the estimated base pair length for each locus, and genotypes were assigned based on the combination of these nine STRs. Isolates were considered identical genotypes if they shared the same alleles across all nine STR markers. Isolates were considered a cluster if there was a single STR marker difference between them. A difference in two STR markers indicated genetic relatedness, while a difference in three or more STR markers were considered unrelated, as previously described [27,28].

Cluster analysis was performed using R version 3.4.4 (R Foundation for Statistical Computing, Vienna, Austria), employing the unweighted pair-group method with arithmetic mean (UPGMA) as previously described [16]. Genotypic diversity was calculated using Simpson’s Diversity Index [29] and visualised alongside clinical outcome using a minimum spanning tree (MST) generated by BioNumerics v.7.6.3 (Applied Maths, Sint-Martens-Latem, Belgium).

### 2.5. Clinical Data

Patient data were collected, including demographics; underlying disease; comorbidities; details of any transplantation; cytomegalovirus (CMV) serostatus; medications (including immunosuppressant and antifungal agents); details of other infections and radiology, microbiology, cytology, and histology results; lung function tests results; macroscopic findings from bronchoscopy; and details of any intensive care admissions and/or surgical procedures and cause of death (if applicable). Each patient was followed for 12 months after the *Aspergillus fumigatus* collection date or until death, if earlier.

### 2.6. Study Definitions

*Aspergillus* isolation was classified as colonisation, tracheobronchitis, bronchial anastomotic infection, or invasive aspergillosis according to the International Society for Heart and Lung Transplantation (ISHLT) definitions [30]. Active bacterial infection was defined as isolation of a bacterial organism and presence of signs and symptoms requiring intravenous antibiotic therapy [30,31]. Cytomegalovirus infection was defined according to published criteria [32]. Respiratory virus infections were defined as positive PCR results from respiratory specimens together with characteristic respiratory symptoms [30]. Lymphopenia was defined as a lymphocyte count of <0.9 × 10^9^/L in the 90 days prior to *A. fumigatus* isolation. Neutropenia was defined as a neutrophil count of <1.5 × 10^9^/L and hypogammaglobulinaemia was defined as an immunoglobulin G level of <6.0 g/L.

CLAD was defined as a substantial and persistent decline (≥20%) in measured FEV_1_ value from the reference (baseline) value. The baseline value was computed as the mean of the best two post-operative FEV_1_ measurements (taken > 3 weeks apart) [33]. CLAD could be present either as a predominantly obstructive ventilatory pattern, a restrictive pattern, or a mixed obstructive and restrictive pattern that is not explained by other conditions. Acute cellular rejection was defined as biopsy proven rejection. This was classified according to the histological appearance and grading system (A:0 to 4) developed by the Lung Rejection Group of ISHLT [34]. Antibody-mediated rejection (AMR) was defined as definite, probable or possible clinical AMR or sub-clinical AMR according to the presence or absence of donor-specific antigens, histology suggestive of AMR, and C4d staining [35].

The definitions of responses to antifungal therapy for *Aspergillus* colonisation, tracheobronchitis, bronchial anastomotic infection or invasive aspergillosis were as previously published [36,37]. Briefly, eradication of colonisation was defined as negative culture from respiratory specimens during the follow-up period. Recurrence of colonisation was defined as isolation of *A. fumigatus* at least 1 month after the completion of the first course of antifungal therapy. Persistent colonisation was defined as ongoing isolation of *A. fumigatus* despite antifungal therapy. Progression to IA was defined as detection of radiological, histopathological, or microbiological features of invasive disease in a previously colonised patient during the 1-year follow-up period.

A complete response to treatment of IA was defined as complete resolution of all attributable signs and symptoms and >90% improvement of radiographic findings. A partial response was defined as >50% resolution of all attributable signs and symptoms and >50% improvement of radiographic findings. Stable disease was defined as no change from baseline or <50% resolution of all attributable signs and symptoms and <50% improvement of radiographic findings. Progressive disease was defined as worsening of signs and symptoms and radiographic findings. A favourable response was defined as a complete or partial response and an unfavourable response was defined as a stable response or progressive disease. Fungal infection-attributable mortality was defined as death with stable or progressive proven or probable fungal pneumonia, tracheobronchitis, or bronchial anastomotic infection at the time of death or with a partial response to antifungal therapy but death occurring as a result of an event involving any of the sites of the original proven, or probable pneumonia, tracheobronchitis, or bronchial anastomotic infection or of an unknown cause or directly due to antifungal toxicity [38].

### 2.7. Statistical Analysis

Descriptive statistics including proportion, mean ± standard deviation (SD), median, range and interquartile range (IQR) were used to describe the data. Comparison of proportions was performed using the chi-squared test or Fisher’s Exact test, as appropriate. Continuous variables were assessed for normality and those that were normally distributed were analysed using the student *t*-test. The Mann–Whitney U test was used to compare non-normally distributed continuous variables. Logistic regression analysis was used to determine risk factors for developing IA in the LTx recipients. Kaplan–Meier curves were used to analyse survival in those with colonisation and IA, CLAD vs. no-CLAD over the two time periods and compared using the log-rank test. In all analyses, a *p*-value of <0.05 was considered statistically significant. Descriptive statistics, comparisons of proportions, and comparisons of continuous variables were performed using GraphPad Prism version 7.04 for Windows (GraphPad Software Inc., La Jolla, CA, USA). Logistic regression analyses and Kaplan–Meier curves were performed using R version 3.4.4 (R Foundation for Statistical Computing).

## 3. Results

### 3.1. Isolates

Of the 60 isolates in this study, eight were removed from the final analysis, as (i) two isolates failed to grow on SCG agar plates; (ii) four were identified as *Aspergillus* species other than *A. fumigatus sensu stricto*; (iii) one failed to amplify all STR regions for genotype analysis; and (iv) one isolate did not have the associated clinical data for classification. One patient had two distinct *A. fumigatus* strains repeatedly identified [16]. This resulted in 53 isolates from 52 separate patients for the final analysis.

### 3.2. Patient Characteristics and Classification of A. fumigatus Isolation

The clinical characteristics are shown in Table 1(a, b). Twenty-five (48.1%) patients first isolated *A. fumigatus* between 1 November 2006 and 31 March 2009. Of these, 12 (48%) and 13 patients (52%) were post LTx or had chronic lung disease, respectively. Six of the 13 (46.2%) with chronic lung disease subsequently underwent LTx at a median (interquartile range [IQR]) of 1141 (385.5–1697.5) days. All 27 patients from the time period of 1 November 2015 to 30 June 2017 were post LTx at the time of first isolation of *A. fumigatus*. The median time (IQR) from LTx to *A. fumigatus* isolation was 180 (26.25–967.3) days.

Overall, CF was the most common underlying disease (18/52 [34.6%]), whereas COPD was most common in LTx recipients (13/39 [33.3%]) (Table 1(a, b)). Transplantation for interstitial lung disease (ILD] occurred exclusively in the later time period (1 November 2015–30 June 2017), and COPD was significantly more common in the earlier time period (1 November 2006–31 March 2009) (*p* = 0.03) (Table 1(b)).

The most common immunosuppressant agents administered to the 39 LTx recipients overall were prednisolone (36/39 [92.3%]), tacrolimus (32/39 [82.1%]), and azathioprine (21/39 [53.8%]) (Table 1(b)). Basiliximab was used for induction in 7/39 (17.9%) of the LTx recipients but only in the later time period (1 November 2015–30 June 2017) (*p* = 0.06).

There was a significant difference in the number of cases of prior lymphopenia between the two time periods (*p* < 0.01) (Table 1(a)), which disappeared when only LTx recipients were compared (*p* = 0.33) (Table 1(b)). The association between prior lymphopenia and IA was further explored by comparing IA and colonised patient data. Significantly more patients with IA were lymphopenic as compared with colonised patients (12/16 [75%] vs. 13/36 [36.1%]; *p* = 0.01).

Colonisation was the most common phenotype associated with *A. fumigatus* isolation (36/52 [69.2%]), followed by IA (10/52 [19.2%]) (Table 2). There was no significant difference in the time from transplant to *A. fumigatus* isolation between colonised and IA patients (180 [21.8–698] days and 149 [36.8–1671] days; *p* = 0.71). No cases of IA were seen in chronic respiratory disease patients (i.e., not transplanted at time of *A. fumigatus* isolation). Comparison of the classification of *A. fumigatus* infections between time periods revealed no significant differences over time (Table 2).

### 3.3. Rejection Episodes in Lung Transplant Recipients

Six of the thirty-nine (15.4%) LTx recipients were diagnosed with acute rejection. No significant difference was found between the proportion of acute rejection episodes that occurred before as compared with after *A. fumigatus* isolation (2/20 vs. 4/19, respectively; *p* = 0.41). Overall, 9/39 (23.1%) LTx developed CLAD; 5/9 (55.6%) in the three months prior to and four (44.4%) in the twelve months after *A. fumigatus* isolation, respectively (*p* = 0.57). LTx recipients with CLAD isolated *A. fumigatus* significantly later after transplantation as compared with those without CLAD (*p* = 0.02).

### 3.4. Co-Infection

Bacterial infections were frequent and the most common type of co-infection (Table S2). There was no significant difference in the infection rates in relation to the timing of *A. fumigatus* isolation; however, a trend toward a greater incidence of CMV reactivation after *A. fumigatus* isolation was observed (0 vs. 5; *p* = 0.06) (Table S2).

### 3.5. Genotypic Diversity of A. fumigatus Isolates

The STR*Af* strain typing detected 45 genotypes among the 53 isolates (52 patients). The M3 marker combination displayed the highest discriminatory power, with M4 displaying the lowest (Table S3). The discriminatory power using both M2 and M3 marker sets was comparable to using a combination of M2, M3 and M4 (Table S3).

**Figure 1:**
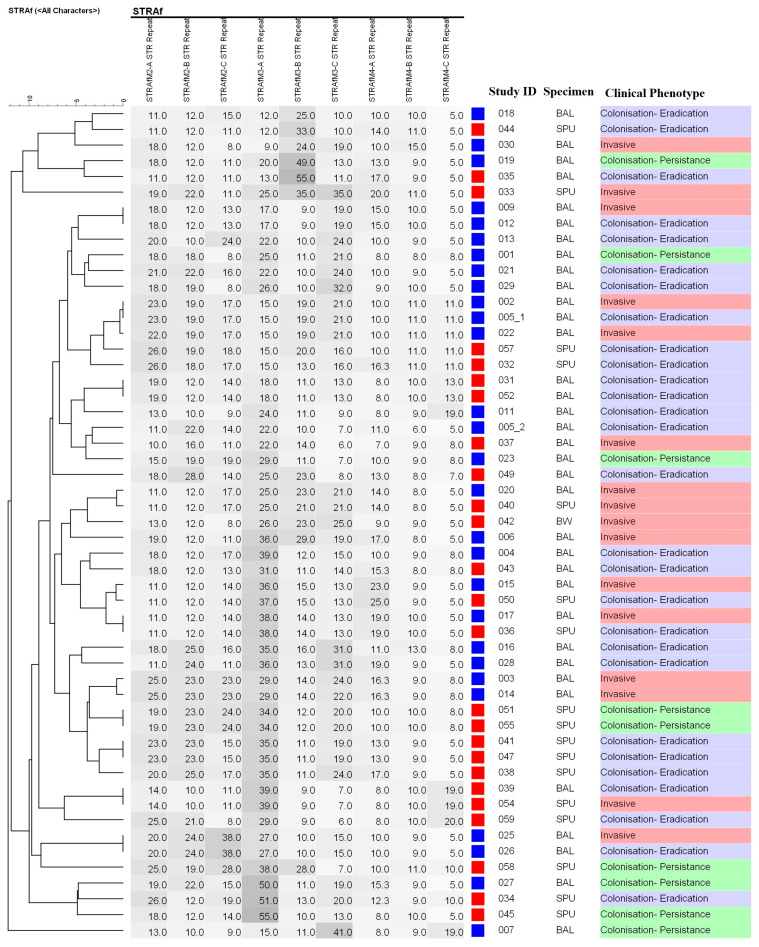
Dendrogram of the Genotype Profiles of the *Aspergillus fumigatus* Isolates from Lung Transplant Recipients or Patients with Chronic Lung Disease. The dendrogram visually illustrates the hierarchical relationships between genotypes and shows the best way to allocate genotypes to clusters. Isolates between 1 November 2006 and 31 March 2009 are represented by the blue squares, and isolates between 1 November 2015–30 June 2017 are represented by the red squares. The deeper the grey colour the greater the number of STR repeats at each locus. Multiple *Aspergillus fumigatus* genotypes from the same patient are denoted by −1 and −2 following study identification number. Tracheobronchitis and bronchial anastomotic infections are classified as invasive [39]. Clusters (isolates differing by 1 STR marker) were detected (C1: 003 and 014; C2: 015 and 050; C3: 020 and 040 and C4: 022, 002 and 005-1). BAL, bronchoalveolar lavage; ID, identification number; SPU, sputum.

Initial STR*Af* typing revealed two isolates (003 and 005; Figure 1) with multiple peaks indicative of either diploid strains, a mixture of separate strains, or an error. To resolve this, the isolates were regrown on SCG agar plates with five individual colonies collected from each. DNA was re-extracted from the individual colonies and STR typing was performed again. All colonies from isolate 003 showed only one genotype, indicating an error in the initial STR*Af* typing experiments. Two distinct genotypes were detected from all colonies for the other isolate (005), indicating a mix of two strains (005_1 and 005_2; Figure 1). The remaining isolates (n = 51) displayed single genotypes on initial typing.

Overall, identical genotypes were seen in 16 *A*. *fumigatus* isolates, with no more than two isolates sharing the same genotype (Figure 1); consequently, there was no dominant genotype. Fourteen of the 16 (87.5%) isolates with identical genotypes were from the same time period as their identical counterpart. Comparing BAL collection dates between isolates with identical genotypes revealed that 10 isolates were collected within two months of each other, while six isolates were collected four months, eight months, and eight years apart. Overall, four clusters (differing by 1 STR marker) (Figure 1) were observed, with the largest cluster (n = 3) containing *A. fumigatus* isolates from three LTx patients who isolated them over an eight-month time period (Figure 1). Isolates from two chronic respiratory patients shared identical genotypes. All others isolated from chronic respiratory patient were unrelated. There was no clustering of genotypes according to time period, indicating that no shifts in the overall *Aspergillus* population occurred over time (Figure 1).

### 3.6. Relationship between Strain Type and Clinical Characteristics

**Figure 2:**
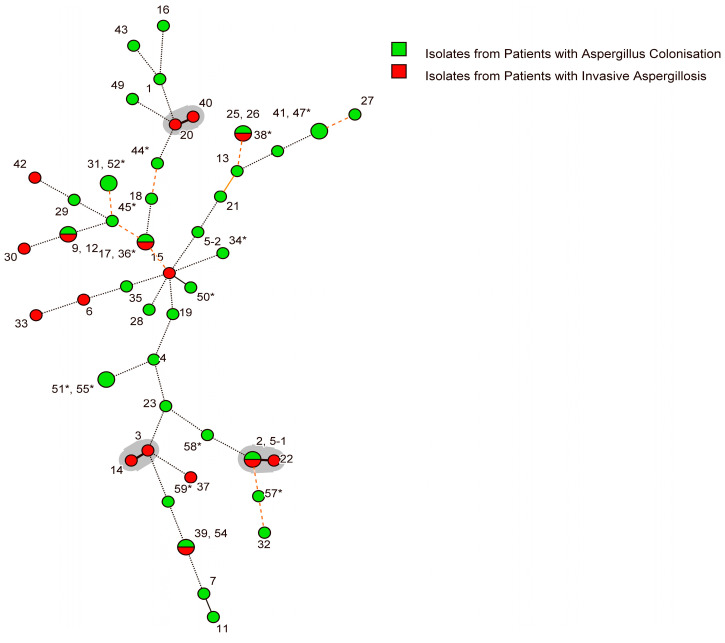
Minimum spanning tree (MST) Showing the Genetic Relatedness of *Aspergillus fumigatus* Isolates from Lung Transplant Recipients or Patients with Chronic Lung Disease. The minimum spanning tree visually estimates the relationship between the different genotypes. The greater the distance between genotypes, the greater the dissimilarity. Tracheobronchitis and bronchial anastomotic infections are classified as invasive aspergillosis [39]. Circles represent different STRA*f* genotypes with the size representing the number of isolates belonging to each genotype. Numbers refer to the study number of each isolate, with the preceding number (in shared genotypes) belonging to the lower portion of divided circles. Connecting lines indicate the similarity between STR genotypes: thick solid black lines indicate a difference of 1 STR locus (defined as a cluster), thin solid black lines—2 STR loci (defined as related), solid orange lines—3 STR loci (defined as unrelated), dashed orange lines—4 STR loci (defined as unrelated), and dotted black lines—5 or more STR loci differences (defined as unrelated). Grey shaded backgrounds represent a cluster. * Denotes isolates from patients with chronic respiratory disease.

Cluster analysis of genotype and *A. fumigatus* infection type revealed 16 genotypes associated with IA; however, five of these (31.3%) were also seen in colonised patients (Figure 2). Five groups of two patients shared identical isolate genotypes but had different clinical characteristics (Figure 2). Six patients with colonisation formed three groups of two patients with identical genotypes (Figure 2). Three IA-associated clusters (differing by one STR locus) were detected (Figure 2). Three sets of genetically related isolates (differing by two loci) were found (7 and 11; 13 and 21; and 15 and 50). Related genotypes 7 and 11 and 13 and 21were from patients who developed colonisation, whereas the related genotypes 15 and 50 were from patients with different clinical characteristics (IA and colonisation, respectively) (Figure 2).

**Figure 3:**
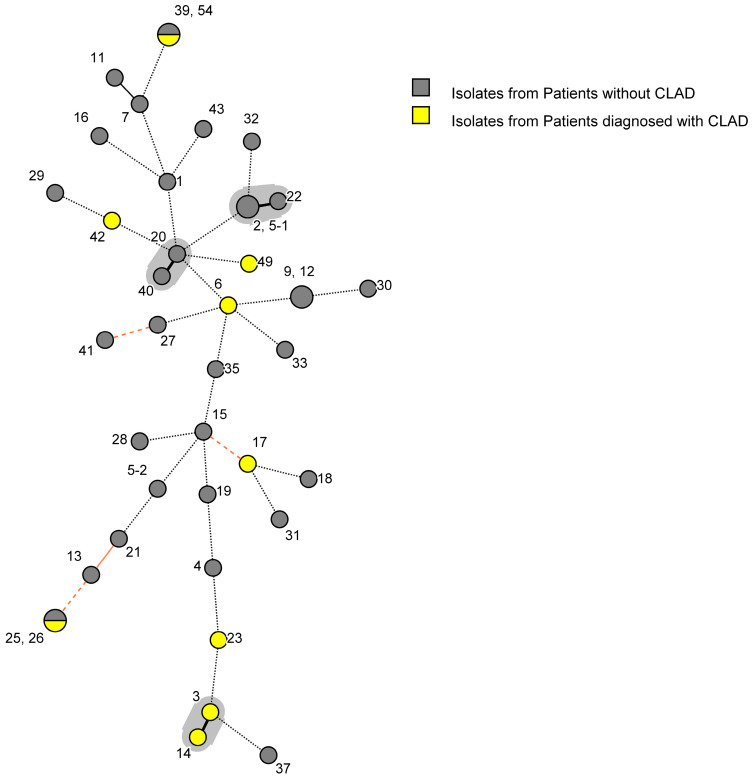
Minimum spanning tree (MST) Displaying the Genetic Relatedness between *Aspergillus fumigatus* Isolates and a Chronic Lung Allograft Dysfunction Diagnosis in the 12 months after *A. fumigatus* Isolation in Lung Transplant Recipients. The minimum spanning tree visually estimates the relationships between the different genotypes. The greater the distance between genotypes, the greater the dissimilarity. Tracheobronchitis and bronchial anastomotic infections are classified as invasive aspergillosis [39]. Circles represent different STRA*f* genotypes, with the size representing the number of isolates belonging to each genotype. Numbers refer to the study number of each isolate, with the preceding number (in shared genotypes) belonging to the lower portions of divided circles. Connecting lines indicate the similarity between STR genotypes: thick solid black lines indicate a difference of 1 STR locus (defined as a cluster), thin solid black lines—2 STR loci (defined as related), solid orange lines—3 STR loci (defined as unrelated), dashed orange lines—4 STR loci (defined as unrelated,) and dotted black lines—5 or more STR loci differences (defined as unrelated). Grey shaded backgrounds represent a cluster.

Cluster analysis of LTx recipients revealed that all nine cases of CLAD had distinct genotypes from one another (Figure 3). Of the nine genotypes, two were shared by non-CLAD patients. One CLAD-associated cluster (containing two isolates) and two non-CLAD-associated clusters (containing two and three isolates, respectively) were identified (Figure 3). Genotypes 7 and 11 displayed genetic relatedness (2 loci difference), as did 13 and 21, with each pair isolated from non-CLAD patients who also had *A. fumigatus* colonisation (Figure 2 and Figure 3).

### 3.7. Antifungal Therapy and Treatment Outcomes

Antifungal drug therapy was administered in 43/52 (82.7%) patients overall. All LTx recipients received treatment, whereas only 4/13 (30.8%) non-LTx chronic respiratory patients were treated with antifungal agents (*p* < 0.01). Voriconazole was the most prescribed drug (24/52 [46.2%]) for a median (IQR) of 49.5 (13–90) days. Posaconazole was the second most common (22/52 [42.3%]), for a median (IQR) of 142.2 (20–264.2) days. Caspofungin and amphotericin B (inhaled) were also used in 4/52 (7.7%) and 3/52 (5.8%) patients. Significantly more voriconazole was administered in the earlier time period (1 November 2006–March 2009) compared with the later time period (1 November 2015–30 June 2017) (*p* = 0.04), while posaconazole treatment was significantly more common in the later time period (*p* < 0.01).

Of those with *A. fumigatus* colonisation, 30/36 (83.3%) had eradicated it within 6 months of isolation. Three of thirty-six (8.3%) cases had a recurrence of *A. fumigatus* colonisation and 2/36 (5.6%) patients persistently isolated *A. fumigatus* over the 12 months of follow-up. The non-transplanted patients with chronic lung disease were significantly more likely to eradicate *A. fumigatus* as compared with LTx recipients at 6 and 12 months of follow-up (*p* = 0.023 and 0.012, respectively) (Table S4). Of those with IA (including tracheobronchitis and bronchial anastomotic infections), 8/16 (50%) had a favourable response to therapy by 12 months of follow-up, with complete responses in 7/16 (43.8%) and a partial response in 1/16 (6.3%).

In the univariate analysis, the only significant risk factor for the development of IA in LTx recipients was *Staphylococcus aureus* infection in the 90 days prior to the index *A. fumigatus* isolation (Table 3). Given the small numbers, multivariate analysis was not performed.

### 3.8. Mortality at 6 and 12 Months Post Aspergillus fumigatus Isolation

All-cause mortality for LTx and non-LTx chronic respiratory patients at 6 months post *Aspergillus fumigatus* isolation was 17.3% (9/52). At 12 months, this increased to 21.2% (11/52). There was no significant difference in all-cause mortality rates between colonised LTx recipients and non-LTx chronic respiratory patients or between the earlier time period as compared with the later time period (Table S4).

Mortality was attributed to IA in 45.5% (5/11) of deaths in the 12 months of follow-up. Of the remaining, two patients died due to a pseudomonal infection, but one had active *A. fumigatus* tracheobronchitis at the time of death. *Strongyloides stercoralis* was implicated in another patient’s death. Two deaths were attributed to CLAD. The remaining death was caused by respiratory and multi-organ failure post LTx.

**Figure 4:**
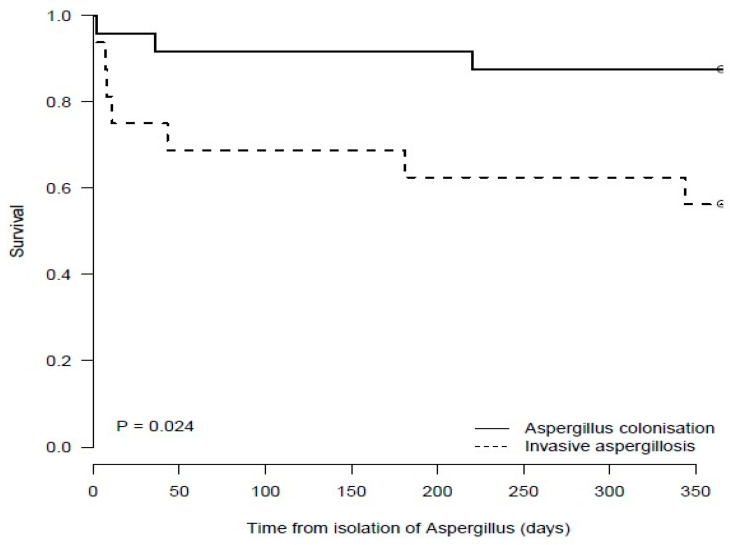
Survival of Lung Transplant Recipients with *Aspergillus fumigatus* Colonisation or Invasive Aspergillosis.

**Figure 5:**
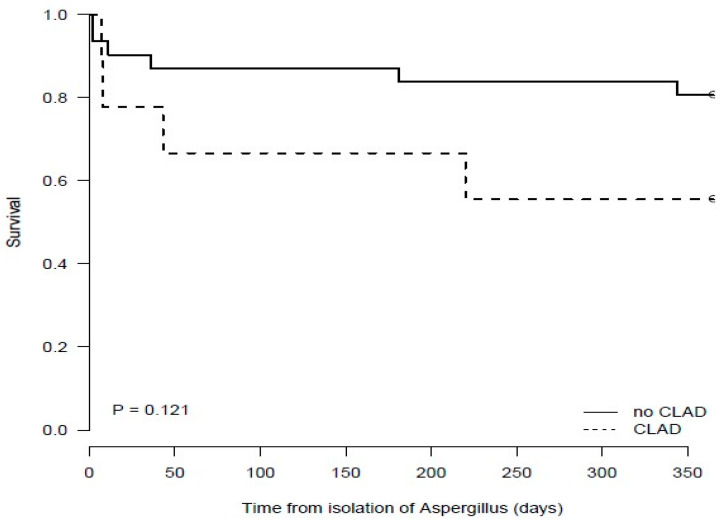
Survival of Lung Transplant Recipients Who Developed Chronic Lung Allograft Dysfunction or Not Post *Aspergillus fumigatus* Isolation.

The median (IQR) time to death from index *A. fumigatus* isolation in colonised patients was 110 days (10.5–211) and was not significantly longer than in IA patients (11 days (7–181)) (*p* = 0.69). Survival was significantly lower in LTx patients who had IA as compared with those patients who were colonised (*p* = 0.024) (Figure 4). In addition, in those that isolated *A. fumigatus,* a trend towards lower survival was seen in LTx patients who had CLAD as compared with those who did not have CLAD (*p* = 0.121) (Figure 5).

## 4. Discussion

This is the first study to examine the genetic variation of *A. fumigatus* in a well-characterised population. Whilst we found several clusters and related strains that were associated with IA and colonisation in the absence of CLAD, no specific genotype–phenotype link was detected. We did, however, find a link between IA and host factors such as prior *S. aureus* infection and lymphopenia in LTx recipients. Importantly, we found that IA in LTx recipients is still associated with significantly lower survival than *A. fumigatus* colonisation.

Our study revealed a high genetic diversity amongst *A. fumigatus* strains (45 genotypes in 52 patients). STR*Af* microsatellite genotyping has previously been shown to be highly discriminatory for investigating genetic diversity [16]. Our findings were consistent, with a D-value of between 0.9819 and 0.9942. The M3 marker displayed the greatest discriminatory power (Table S3), as previously described by de Valk et al. [16]. Identical strains were found in different patients, which could represent nosocomial acquisition of *A. fumigatus*. Prior studies have shown that environmental strain types can persist within the hospital [40]. We detected identical strains over several months to years, making nosocomial acquisition in our cohort unlikely. We did not detect a dominant genotype, indicating that patient-to-patient transmission and acquisition from a common source were unlikely, consistent with Vanhee et al. [18]. However, the main aim was not to detect transmission, and the study was not designed accordingly.

STR*Af* genotyping has previously been used to determine the relationship between strain type and clinical characteristics with conflicting results. De Valk et al. determined that multiple genotypes were isolated from the respiratory tract, but only a single genotype was isolated from the sites of dissemination (e.g., kidney, heart, spleen). This indicates that one genotype becomes dominant and invades the surrounding lung tissues and then disseminates to other organs [19]. Vanhee et al. found that only certain genotypes were persistently isolated from the respiratory tract of CF patients [18]. Persistent colonisation has been associated with an increased risk of progression to IA [4]. In contrast, Escribano et al. used STR*Af* typing on *Aspergillus* isolates from 236 patients and determined that it could not discriminate between isolates from IA patients and colonised patients [41]. They did not collect detailed clinical data to adequately define their patients, likely confounding their findings [41]. Our study aimed to evaluate the utility of this strain-typing method for differentiating between IA and colonising strains of *A. fumigatus* in a well-defined population. Additionally, we used this method to investigate the association between strain type and CLAD, which had not been explored previously. Overall, we found no clear association between genotype and IA or CLAD. Strains in our population were largely unrelated, and clinical characteristics were variable between the strains that were related. We did, however, find a cluster (differing by 1 locus) of *A. fumigatus* isolates associated with IA and related isolates (differing by 2 loci) associated with colonisation in the absence of CLAD, indicating that such a relationship likely exists.

Several studies have detected strain-dependent virulence factors in *Drosophila* and *Galleria mellonella* models of IA and in isolates from patients with pulmonary aspergilloma and chronic-necrotising pulmonary aspergillosis using whole genome sequencing (WGS) [42,43,44]; however, we could not conclusively detect this in our study using STR typing. STR typing has shown utility in population genetics, local disease epidemiology, and route-of-transmission studies [45]. In addition, STR*Af* only detects predetermined sections of the genome, whereas WGS captures the whole genome, indicating the latter is likely to be much more sensitive. Ballard, et al. and Hagiwara, et al. demonstrated that WGS could detect in-host microevolution throughout the course of *Aspergillus* infection, whereas STR typing lacked the discriminatory power for this [44,46]. Our data would indicate that STR*Af* typing is not the optimal method for cohort strain-type virulence studies and that the more sensitive WGS should be used in the future to examine the relationships between different strains and different clinical characteristics/phenotypes.

An association between *A. fumigatus* colonisation and CLAD has previously been shown [22]. In addition, small conidia (≤3.5 μm) *Aspergillus* species are associated with a greater risk of precipitating CLAD [23]. In our cohort of *A. fumigatus* (a small conidia-producing species), some genotypes were associated with CLAD and others were not; however, no significant association was found. Further analysis is required using WGS in a larger cohort who are followed over a longer period to determine if certain strains have the propensity to precipitate CLAD. Such information would be very valuable in the development of new strategies for the management of *Aspergillus* isolation to prevent CLAD.

Like other studies, we noted that host–immunity and host–pathogen interactions play a central role in the development of IA. We observed that IA only occurred in LTx recipients and that lymphopenia in the 90 days prior to *A. fumigatus* isolation was more frequent in IA LTx patients as opposed to colonised LTx patients. CMV reactivation has long been considered a risk factor for developing aspergillosis due to the immunomodulatory effects of CMV [47]. We observed an increase in CMV reactivation in LTx recipients following *A. fumigatus* isolation. Prior *Aspergillus* isolation has not previously been shown to be a risk factor for CMV infection or disease. Little is known about the direct interaction between *A. fumigatus* and *S. aureus* in LTx recipients. Our study showed that prior isolation of *S. aureus* is a significant predictor for the development of IA in LTx recipients. Animal studies have demonstrated that *S. aureus* causes acute lung injury by increasing chemokines to recruit neutrophils [48]. The acute lung injury may provide a portal of entry for *Aspergillus* invasion.

Several changes in LTx selection criteria, immunosuppressive regimens and antifungal therapy over time have been detected in this study; however, we saw no significant changes in mortality. This may reflect a reliance on culture for diagnosing and guiding the treatment of *A. fumigatus* infections. Newer diagnostic assays, including *Aspergillus* galactomannan (GM) and PCR, are now available. Husain et al. used GM in addition to cultures to classify fungal infections in LTx recipients and guide antifungal therapy. They found that 48.2% of LTx recipients had mould colonisation, and 27.5% had IA [3]. Over 50% of the cases of IA occurred in those who did not have a prior positive culture. In our study we found a statistically significant lower rate of survival in IA cases compared with colonised cases (Figure 4), whereas Husain et al. found that patients who had IA or were given pre-emptive therapy (directed by a positive GM result) had similar hazards of mortality at 1 year as compared with patients who were culture or GM negative [3]. Our outcomes with *Aspergillus* infection indicate that a new diagnostic algorithm that incorporates the more sensitive diagnostic assays for earlier and more accurate diagnosis is warranted.

This study has several limitations, including the small sample size and retrospective study design. These limited the power to detect differences in clinical manifestations according to strain variation and may have introduced bias into the clinical classifications and subsequent analysis. Collection of clinical data was particularly beneficial in providing clinical context for the accompanying molecular data, but the power of our epidemiological analyses was likely impaired by the low numbers of patients involved in the study. While associations were found between certain strain-type and clinical characteristics and outcomes, the small numbers in each group affected the certainty of the results and need to be explored further in a larger group of patients. This study did not include a control group consisting of patients without *A. fumigatus* isolation, so conclusions drawn from risk-factor analyses, mortality rates, and prevalence of CLAD may be biased. Although prior studies have indicated that the median onset time for CLAD was less than 12 months post *A. fumigatus* infection [22], following these patients for a longer time period may have been helpful to elucidate clinical outcomes, particularly CLAD, and this should be included in the methodology of future cohort studies. In the present study, we did not examine sequential isolates from patients, so we cannot be certain that the genotypes that we identified as associated with colonisation will not cause IA in the same patient at a later date. Further studies examining this are required.

## 5. Conclusions

A strong and specific link between certain *A. fumigatus* strains and certain clinical manifestations was not found in this study. However, several clusters and related strains were detected with an association with IA and colonisation in patients who did not have CLAD. Further analyses using fungal WGS are required to explore this link. Mortality rates in those who isolated *A. fumigatus* remained static over time despite changes in LTx procedures, indicating the importance of early diagnosis of IA. Our data reinforce the concept that immune function and other infections have a major impact on the development of IA. We detected a trend toward a greater incidence of CMV reactivation post *A. fumigatus* isolation in lung transplant recipients. This indicates that *A. fumigatus* may have immunomodulatory effects. Overall, further research is required to determine the pathogenicity and immunogenicity of specific *A. fumigatus* strains.

## Figures and Tables

**Table 1:** (a) Baseline Characteristics of Both Lung Transplant and Chronic Respiratory Disease Patients who isolated *Aspergillus fumigatus* Across the Time Periods of 1 November 2005–31 March 2009 and 1 November 2015–30 June 2017. (b) Baseline Characteristics of Lung Transplant Patients Only who isolated *Aspergillus fumigatus* Across the Time Periods of 1 November 2005–31 March 2009 and 1 November 2015–30 June 2017.

(a)					
**Characteristic**		**Overall** **N: 52 (%)**	**1 November 2006–31 March 2009** **N: 25 (%)**	**1 November 2015–30 June 2017** **N: 27 (%)**	***p*** **-Value ^1^**
Age in years, median (IQR) ^2^		47 (29.0)	40 (21.0)	53 (32.5)	0.07
Sex (Female)		27 (51.9)	15 (60.0)	12 (44.4)	0.27
Underlying disease					
	COPD	15 (28.8)	9 (36.0)	6 (22.2)	0.28
	CF	18 (34.6)	12 (48.0)	6 (22.2)	0.05
	Interstitial Lung Disease	4 (7.7)	0 (0.0)	4 (14.8)	0.11
	Non-CF Bronchiectasis	3 (5.8)	1 (4.0)	2 (7.4)	0.61
	Alpha-1 Antitrypsin Deficiency	3 (5.8)	0 (0.0)	3 (11.1)	0.24
	Pulmonary Hypertension	5 (9.6)	0 (0.0)	5 (18.5)	0.05
	Other ^3a^	4 (7.7)	3 (12.0)	1 (3.7)	0.26
Comorbidities ^4^					
	Diabetes mellitus	17 (32.7)	8 (32.0)	9 (33.3)	0.92
	Insulin Dependent	14 (26.9)	5 (20.0)	9 (33.3)	0.29
	Oral hypoglycaemics only	3 (5.8)	3 (12.0)	0 (0.0)	0.07
	Chronic renal failure	30 (57.7)	8 (32.0)	22 (81.5)	<0.001
	Lymphopenia	25 (48.1)	7 (28.0)	18 (66.7)	<0.01
	Neutropenia	4 (7.7)	0 (0.0)	4 (14.8)	0.11
	GORD	16 (30.8)	9 (36.0)	7 (25.9)	0.44
	Hypogammaglobinaemia	5 (9.6)	1 (4.0)	4 (14.8)	0.19
(b)					
**Characteristics of Lung Transplant Recipients**		**Overall** **N: 39 (%)**	**1 November 2006–31 March 2009** **N: 12 (%)**	**1 November 2015–30 June 2017** **N: 27 (%)**	***p*** **-Value ^1^**
Age in years, median (IQR) ^2^		50 (30.0)	49 (16.0)	50 (33.0)	0.41
Sex (Female)		19 (48.7)	7 (58.3)	12 (44.4)	0.43
Underlying disease					
	COPD	13 (33.3)	7 (58.3)	6 (22.2)	0.03
	CF	9 (23.1)	3 (25.0)	6 (22.2)	0.85
	Interstitial Lung Disease	4 (10.3)	0 (0.0)	4 (14.8)	0.28
	Non-CF Bronchiectasis	2 (5.1)	0 (0.0)	2 (7.4)	1
	Alpha-1 Antitrypsin Deficiency	3 (7.7)	0 (0.0)	3 (11.1)	0.54
	Pulmonary Hypertension	5 (12.8)	0 (0.0)	5 (18.5)	0.15
	Other ^3b^	3 (7.7)	2 (16.7)	1 (3.7)	0.16
Co-morbidities ^4^					
	Diabetes mellitus	14 (35.9)	5 (41.6)	9 (33.3)	0.62
	Insulin Dependent/Requiring	13 (33.3)	4 (33.3)	9 (33.3)	1
	Oral hypoglycaemics only	1 (2.6)	1 (8.3)	0 (0.0)	0.13
	Chronic renal failure	29 (74.4)	7 (58.3)	22 (81.5)	0.13
	Lymphopenia	24 (61.5)	6 (50.0)	18 (66.7)	0.33
	Neutropenia	4 (10.3)	0 (0.0)	4 (14.8)	0.28
	GORD	12 (30.8)	5 (41.6)	7 (25.9)	0.33
	Hypogammaglobinaemia	5 (12.8)	1 (8.3)	4 (14.8)	0.58
Type of transplant					
	BSLT	35 (89.7)	8 (66.6)	27 (100)	<0.01
	SLT	4 (10.3)	4 (33.3)	0 (0.0)	<0.01
Pre-transplant *Aspergillus* isolation		2 (5.1)	1 (8.3)	1 (3.7)	1
Immunosuppressants ^5,6^					
	Cyclosporin	8 (20.5)	6 (50.0)	2 (7.4)	<0.01
	Methylprednisolone	11 (28.2)	0 (0.0)	11 (40.7)	0.01
	Mycophenolate	15 (38.5)	4 (33.3)	11 (40.7)	0.66
	Tacrolimus	32 (82.1)	6 (50.0)	26 (96.3)	<0.01
	Prednisolone	36 (92.3)	11 (91.7)	25 (92.6)	0.92
	Azathioprine	21 (53.8)	6 (50.0)	15 (55.6)	0.75
	Everolimus	1 (2.6)	1 (8.3)	0 (0.0)	0.13
	Basiliximab	7 (17.9)	0 (0.0)	7 (25.9)	0.06

Data presented as number (%) unless otherwise stated. ^1^ *p*-value is for difference between the two time periods (1 November 2006–31 March 2009 and 1 November 2015–30 June 2017). ^2^ At time of *Aspergillus fumigatus* isolation. ^3a^ Other underlying diseases include X-linked agammaglobulinemia (n = 1); lymphangioleiomyomatosis (n = 1); pulmonary fibrosis (n = 1); bronchiolitis (n = 1). ^3b^ Other underlying diseases include X-linked agammaglobulinemia (n = 1); lymphangioleiomyomatosis (n = 1); bronchiolitis (n = 1); ^4^ Had one or more comorbidities. ^5^ On one or more immunosuppressant. ^6^ No patient received anti-thymocyte globulin, sirolimus, or dexamethasone; BSLT, bilateral sequential lung transplant; CF, cystic fibrosis; COPD, chronic obstructive pulmonary disease; GORD, gastro-oesophageal reflux disease; IQR, interquartile range; N, number; SLT, single lung transplant.

**Table 2:** Classification of *Aspergillus fumigatus* Isolation.

Category	Overall N: 52 (%)	Lung Transplant N: 39 (%)	Non-LT CRD N: 13 (%)	*p*-Value ^1^	1 November 2006–31 March 2009 N: 25 (%)	1 November 2015–30 June 2017 N: 27 (%)	*p*-Value ^2^
Colonisation	36 (69.2)	23 (58.9)	13 (100.0)	<0.01	20 (80.0)	16 (59.3)	0.11
TB/BA infection	6 (11.5)	6 (15.4)	0 (0.0)	0.14	1 (4.0)	5 (18.5)	0.11
IA/*Aspergillus* Pneumonia Proven Probable	10 (19.2) 7 (13.5) 3 (5.8)	10 (25.6) 7 (17.9) 3 (7.7)	0 (0.0) 0 (0.0) 0 (0.0)	0.04	4 (16.0) 3 (12.0) 1 (4.0)	6 (22.2) 4 (14.8) 2 (7.4)	0.57

^1^ *p*-value for difference between lung transplant and non-lung transplant other chronic respiratory disease. ^2^ *p*-value for difference between the two time periods; 1 November 2006–31 March 2009 and 1 November 2015–30 June 2017. CRD, chronic respiratory disease; IA, invasive aspergillosis; LT, lung transplant; N, number; TB/BA, tracheobronchitis/bronchial anastomotic infection.

**Table 3:** Univariate Analysis of Risk Factors for Invasive Aspergillosis in Lung Transplant Recipients.

Variable		Exposure N: 39 (%)	OR (95% CI)
Age			
	12 to <40	13 (33.3)	Reference
	≥40 to <50	7 (17.9)	0.9 (0.1–6.57)
	≥50 to <60	9 (23.1)	1.12 (0.17–7.06)
	≥60 to 70	10 (25.6)	3.94 (0.76–24.03)
Sex (Female)		19 (48.7)	0.56 (0.15–1.98)
Underlying disease			
	CF	9 (23.1)	Reference
	COPD	13 (33.3)	1.71 (0.3–11.1)
	Other ^1^	17 (43.6)	1.27 (0.24–7.62)
Pre-transplant *Aspergillus*		2 (5.1)	1.53 (0.06–40.8)
Time period			
	1 November 2006–31 March 2009	12 (30.8)	Reference
	1 November 2015–30 June 2017	27 (69.2)	1.1 (0.29–4.48)
Diabetes		14 (35.9)	1.56 (0.42–5.83)
Lymphopenia		24 (61.5)	3.00 (0.79–13.24)
GORD		12 (30.8)	1.10 (0.27–4.37)
Hypogammaglobulinaemia		5 (12.8)	0.33 (0.22–2.55)
Chronic kidney disease		29 (74.4)	2.17 (0.51–11.45)
Immunosuppressive agents			
	Cyclosporin	8 (20.5)	4.20 (0.91–23.35)
	Mycophenolate	15 (38.5)	1.00 (0.26–3.69)
	Tacrolimus	32 (82.1)	0.60 (0.12–2.96)
	Prednisolone	36 (92.3)	1.36 (0.12–30.91)
	Methylprednisolone	11 (28.2)	1.36 (0.32–5.63)
	Azathioprine	21 (53.8)	1.09 (0.3–3.97)
	Basiliximab	7 (17.9)	2.33 (0.44–13.59)
	Everolimus	1 (2.6)	NA
Other infection			
	*Pseudomonas aeruginosa*	6 (15.4)	0.71 (0.09–4.2)
	*Staphylococcus aureus*	7 (17.9)	13.8 (2.01–279.23)
	CMV	0 (0)	NA
	Respiratory viruses	2 (5)	NA
Rejection			
	*Acute*	6 (1.35)	1.53 (0.06–40.8)
	*Chronic*	9 (23.1)	3.67 (0.62–29.36)
Time from Tx to *Aspergillus* isolation, days			
	1 to <30.75	9(25)	Reference
	≥30.75 to <180	10 (25)	2.33 (0.38–16.28)
	≥180 to <803.75	10 (25)	1.00 (0.14–7.16)
	≥803.75 to <3314	10 (25)	2.33 (0.38–16.28)

^1^ Other Underlying Diseases Include interstitial lung disease (n = 4), non-CF bronchiectasis (n = 2), alpha-1 antitrypsin deficiency (n = 3), pulmonary hypertension (n = 5), X-linked agammaglobulinemia (n = 1), lymphangioleiomyomatosis (n = 1), bronchiolitis (n = 1). CF, cystic fibrosis; CI, confidence interval; COPD, chronic obstructive pulmonary disease; GORD, gastro-oesophageal reflux disease; OR, odds ratio; Tx, transplant.

## Data Availability

Data are included in the tables and figures, herein and additionally, for research purposes only and are available upon reasonable request.

## Supplementary Materials

The following supporting information can be downloaded at https://www.mdpi.com/article/10.3390/jof10020095/s1. Table S1. STRA*f* Genotyping Multiplex PCRs and Corresponding Primer Sets [16]; Table S2. Bacterial and Viral Co-Infections; Table S3. Discriminatory Power for Each STRA*f* Marker and Their Combinations; Table S4. Clinical Outcomes 6- and 12-months post-*Aspergillus fumigatus* Isolation.
