# Supplementary material for: Multi-Locus Microsatellite Typing of Colonising and Invasive Aspergillus fumigatus Isolates from Patients Post Lung Transplantation and with Chronic Lung Disease
Source: J Fungi (Basel). 2024 Jan 24;10(2):95. doi: 10.3390/jof10020095 (PMC10889758; doi:10.3390/jof10020095)
Supplement: Supplementary file 1 [file jof-10-00095-s001.zip › jof-2810619-supplementary.pdf]

Journal of Fungi

Table S1. STR*Af* Genotyping Multiplex PCRs and Corresponding Primer Sets [16]

Multiplex PCR Name		Sequence (5′ to 3′)	
		**Forward**	**Reverse**
M2			
	STR*Af*-2A	6FAM-AAGGGTTATGGCCATTAGGG	GACCTCCAGGCAAAATGAGA
	STR*Af*-2B	VIC-TATTGGATCTGCTCCCAAGC	GAGATCATGCCCAAGGATGT
	STR*Af*-2C	NED-TCGGAGTAGTTGCAGGAAGG	AACGCGTCCTAGAATGTTGC
M3			
	STR*Af*-3A	6FAM-GCTTCGTAGAGCGGAATCAC	GTACCGCTGCAAAGGACAGT
	STR*Af*-3B	VIC-CAACTTGGTGTCAGCGAAGA	GAGGTACCACAACACAGCACA
	STR*Af*-3C	NED-GGTTACATGGCTTGGAGCAT	GTACACAAAGGGTGGGATGG
M4			
	STR*Af*-4A	6FAM-TTGTTGGCCGCTTTTACTTC	GACCCAGCGCCTATAAATCA
	STR*Af*-4B	VIC-CGTAGTGACCTGAGCCTTCA	GGAAGGCTGTACCGTCAATCT
	STR*Af*-4C	NED-CATATTGGGAAACCCACTCG	ACCAACCCATCCAATTCGTAA

Table S2. Bacterial and Viral Co-Infections

Type of Infection	Pre-*Aspergillus* Isolation[1] N: 52 (%)	Post-*Aspergillus* isolation[2] N: 52 (%)	p-value[3]	Pre- and post-*Aspergillus* isolation episodes in LT-recipients N: 78 (%)[4]	Pre- and post-*Aspergillus* isolation episodes in non-LT recipients N: 26 (%)[4]	p-value[5]
Bacterial						
Pseudomonas spp.	15 (28.8)	13 (25.0)	0.66	12 (15.4)	16 (61.5)	<0.01
S. aureus	7 (13.5)	11 (21.2)	0.30	16 (20.5)	3 (11.5)	0.31
Other gram positive[6]	1 (1.9)	5 (9.6)	0.09	5 (6.4)	1 (3.8)	0.62
Other gram negative[7]	7 (13.5)	6 (11.5)	0.76	11 (14.1)	2 (7.7)	0.40
Viral						
CMV	0 (0.0)	5 (9.6)	0.06	5 (6.4)	0 (0.0)	0.33
RVI[8]	2 (3.8)	5 (9.6)	0.24	6 (7.7)	1 (3.8)	0.19

[1]Defined as isolation of another organism in the 90 days prior to the index *A. fumigatus* isolation. [2]Defined as isolation of another organism in the 90 days post the index *A. fumigatus* isolation. [3]P-value for difference between pre-*A. fumigatus* and post-*A. fumigatus* isolation of another organism. [4]Represents the time-periods of 90 days pre- plus the 90 days post-*Aspergillus fumigatus* isolation. [5]P-value for difference between lung transplant and non-lung transplant chronic respiratory disease patients. [6]Other gram-positive organisms: Pre-*Aspergillus fumigatus* isolation: *Staphylococcus cohnii* (n=1). Post-*Aspergillus fumigatus* isolation: *Enterococcus faecium* (n=3), *S. epidermidis* (n=1), *Streptococcus pneumoniae* (n=1). [7]Other gram-negative organisms: Pre-*Aspergillus fumigatus* isolation: *Enterobacter aerogenes* (n=2), *Stenotrophomonas* spp. (n=2), *Serratia marcescens* (n=2), *Klebsiella pneumoniae* (n=1). Post-*Aspergillus fumigatus* isolation: *Stenotrophomonas* spp. (n=2), *Acinetobacter* spp. (n=2), *Klebsiella pneumoniae* (n=1), *Sphingomonas paucimobilis* (n=1). [8]Respiratory viruses: Pre-*Aspergillus fumigatus* isolation: Influenza A (n=1), Picornavirus (n=1). Post-*Aspergillus fumigatus* isolation: Picornavirus (n=4), Human metapneumovirus (n=1). CMV, cytomegalovirus; RVI, respiratory virus infection; *S. aureus*, *Staphylococcus aureus.*

Table S3. Discriminatory Power for Each STR*Af* Marker and Their Combinations.

**Marker Combination**	**Number of Genotypes**	***D-Value*[1]**
M2	39	0.9869
M3	43	0.992
M4	35	0.9819
M2+M3	45	0.9942
M2+M4	43	0.9927
M3+M4	44	0.9935
M2+M3+M4	45	0.9942

[1]D-value calculated using Simpson's Diversity Index

Table S4. Clinical Outcomes 6- and 12-months post-*Aspergillus fumigatus* Isolation.

Infection type/responses to therapy	Overall<br>N: 52 (%)	Lung Transplant<br>N: 39 (%)	Non-Lung transplant CRD<br>N: 13 (%)	p-value[1]	1 Nov 2006-31 Mar 2009<br>N: 25 (%)	1 Nov 2015- 30 Jun 2017<br>N: 27 (%)	p-value[2]
**Colonisation**							
**6-Months[3]**							
**Responses to therapy**							
Eradication	30 (57.6)	19 (48.7)	11 (84.6)	0.023	18 (72.0)	12 (44.4)	0.15
Persistence	2 (3.8)	2 (5.1)	0 (0.0)	1	0 (0.0)	2 (7.4)	0.49
Recurrence	1 (1.9)	0 (0.0)	1 (7.7)	0.25	1 (4.0)	0 (0.0)	1
Progression to invasive fungal disease	0 (0.0)	0 (0.0)	0 (0.0)	1	0 (0.0)	0 (0.0)	1
All-cause mortality	4 (7.7)	3 (7.7)	1 (7.7)	1	2 (8.0)	2 (7.4)	1
**12-Months[4]**							
**Responses to therapy**							
Eradication	28 (53.8)	17 (43.6)	11 (84.6)	0.012	17 (68.0)	11 (40.7)	0.058
Persistence	2 (3.8)	2 (5.1)	0 (0.0)	1	0 (0.0)	2 (7.4)	0.49
Recurrence	3 (5.8)	2 (5.1)	1 (7.7)	1	2 (8.0)	1 (3.7)	0.60
Progression to invasive fungal disease	0 (0.0)	0 (0.0)	0 (0.0)	1	0 (0.0)	0 (0.0)	1
All-cause mortality	4 (7.7)	3 (7.7)	1 (7.7)	1	2 (8.0)	2 (7.4)	1
**Invasive Aspergillosis[5]**							
**6-Months[3]**							
**Responses to therapy**							
Complete response	7 (13.5)	7 (17.9)	0 (0.0)	-	1 (4.0)	6 (22.2)	0.10
Partial response	1 (1.9)	1 (2.6)	0 (0.0)	-	0 (0.0)	1 (3.7)	1
Stable disease	2 (3.8)	2 (5.2)	0 (0.0)	-	1 (4.0)	0 (0.0)	0.48
Progressive disease	1 (1.9)	1 (2.6)	0 (0.0)	-	0 (0.0)	1 (3.7)	1
All-cause mortality	5 (9.6)	5 (12.8)	0 (0.0)	-	3 (12.0)	3 (11.1)	1

**12-Months[4]**							
**Responses to therapy**							
Complete response	7 (13.5)	7 (17.9)	0 (0.0)	-	1 (4.0)	6 (22.2)	0.10
Partial response	1 (1.9)	1 (2.6)	0 (0.0)	-	0 (0.0)	1 (3.7)	1
Stable disease	1 (1.9)	1 (2.6)	0 (0.0)	-	0 (0.0)	1 (3.7)	1
Progressive disease	0 (0.0)	0 (0.0)	0 (0.0)	-	0 (0.0)	0 (0.0)	1
All-cause mortality	7 (13.5)	7 (17.9)	0 (0.0)	-	4 (16.0)	3 (11.1)	0.69

[1]P-value is for difference between lung transplant and non-lung transplant chronic respiratory disease.[2]P-value is for difference between 1 Nov 2006-31 Mar 2009 and 1 Nov 2015-30 Jun 2017 cohorts.[3]6-months post-*Aspergillus fumigatus* isolation. [4]12-months post-*Aspergillus fumigatus* isolation. [5]Tracheobronchitis and bronchial anastomotic infections classified under invasive aspergillosis [39].
